# Digital citizenship for health: Mapping and evaluation of existing platforms supporting digital, health, and civic literacy among young people

**DOI:** 10.1371/journal.pdig.0000737

**Published:** 2026-07-16

**Authors:** Racheal Oluwabukunmi Ogundipe

**Affiliations:** Independent Researcher, Lagos, Lagos State, Nigeria; Peng Cheng Laboratory, CHINA

## Abstract

Young people often lack access to literacy platforms that address their specific needs, leaving them vulnerable to misinformation, online harm, privacy risks, and limited capacity to engage in digital, health, and civic spaces. This study aimed to identify existing digital citizenship for health (DC4H) platforms and evaluate the extent to which they address the needs of young people. This study was conducted in two phases. The first phase identified relevant platforms through an integrated search approach combining academic databases, web searches, social media queries, and AI-assisted tools. The second phase evaluated the identified platforms using walkthrough assessments and document review based on 13 predefined criteria related to youth-centered values and functional literacy support. A comparative analysis was ultimately used to examine patterns, highlight strengths, and identify gaps across the platforms. The study evaluated 38 existing digital, health, and civic literacy platforms. The analysis revealed significant gaps in current platforms. All evaluated platforms focused on improving knowledge and sharing information, but fewer supported skill development (17). Most of the evaluated platforms lacked multilingual support and alternative learning formats, excluding subgroups of young people, including those with disabilities, limited digital access, and language barriers. In addition, most platforms addressed only one literacy domain rather than integrating digital, health, and civic literacy. There are gaps in existing DC4H platforms that highlight the need for more integrated solutions that connect digital, health, and civic literacy while addressing the identified limitations.

## Author summary

DC4H integrates digital, health, and civic literacy to empower individuals, particularly young people, to navigate digital health ecosystems effectively. This framework promotes equitable access to health information, informed decision-making, and active participation in personal and public health outcomes while addressing critical issues of digital literacy disparities, privacy, and inclusivity. Building young people’s digital citizenship capacity for health goes beyond providing information. It requires strengthening their skills and confidence to engage meaningfully in digital and civic spaces while taking responsibility for their health and well-being. This can be achieved by developing DC4H platforms that reflect the diverse cultural, social, and digital needs of young people worldwide. It also calls for stronger stakeholder commitment to ensure adequate resources are accessible. This research evaluated digital, health, and civic literacy platforms, examined their effectiveness, and identified gaps to inform the development of platforms that better support young people’s DC4H.

## Introduction

Digital transformation is rapidly redefining health and well-being. This concept positions digital technologies as an increasingly important determinant of health, especially for young people [1]. Digital transformation in healthcare involves creating inclusive systems that actively improve access, support health equity, protect digital rights, and empower young people [2]. According to the Lancet and Financial Times Commission on governing health futures 2030, achieving universal health coverage (UHC) through digital health requires high levels of digital, health, and civic literacy. Developing these literacies is specifically important for children and young people, who need the knowledge and skills to manage their health and well-being online, make informed choices, and develop the capabilities to participate in future health and digital economies [1].

To address these needs, digital citizenship for health (DC4H) integrates these literacies into a single framework [3]. Digital literacy provides the skills and knowledge needed to use digital technologies effectively [4]. Health literacy equips young people with the ability to obtain, understand, and use health information to make appropriate and informed decisions [5]. Civic literacy supports one’s participation in civic life by building awareness, a sense of responsibility, and the processes that shape collective decision-making [6]. These literacies are interconnected, each reinforcing the others, creating a foundation for young people to navigate digital spaces safely, make informed health decisions, and take active roles in civic life [3].

However, significant challenges exist in current digital engagement among young people. Studies show that over 90% of young people aged 16–30 have access to a mobile device and the internet [7–9]. Despite growing up in highly digitized and information-dense environments, many young people still lack the comprehensive skills needed for meaningful participation in digital spaces [10]. These skills are particularly important given evidence of health-related challenges arising from technology use, including online bullying, exposure to harmful information, mental health difficulties, and privacy risks [11]. Some of these challenges often lead to severe emotional and psychological trauma, causing the victim to feel anxious and depressed and even affecting their self-esteem [12]. Other challenges, such as exposure to misinformation, may lead to dangerous behaviors, particularly regarding their healthcare [13]. These findings underscore the importance of adequate digital and health literacy among young people.

Civic literacy and engagement are equally central to young people’s development, as they provide young people with a voice to make a difference in community initiatives and to bring about social change [14]. This can strengthen connections between citizens and political representatives by enabling faster communication, real-time feedback, greater transparency, and new opportunities for participation in decision-making [14]. For health, civic engagement in the digital space helps ensure that services are shaped by the needs and experiences of the people it serves. When young people can provide real-time feedback, challenge misinformation, and participate in decision-making, health systems become more transparent and better at addressing issues such as access, equity, and quality of care.

Digital citizenship has gained global attention, with parents, educators, and policymakers developing curricula and digital platforms to equip young people with digital and civic literacy. However, health literacy is often missing from this mix, despite its direct relevance to young people’s well-being and future participation in society. Most discussions focus on online safety, rights, and responsibilities, but introducing health literacy within this framework could better prepare young people. Including health in ‘Digital Citizenship’ would also contribute to achieving UHC while supporting the growth of digitally immersed young people. To achieve this, there is a need to develop platforms that bring together digital, health, and civic literacy. Before creating new platforms, it is essential to understand what already exists to avoid duplication and identify gaps.

Currently, there is little systematic research mapping DC4H platforms globally. The effectiveness of existing platforms, and whether they adequately meet young people’s integrated digital, health, and civic literacy needs, remains unclear. This lack of evidence represents a critical knowledge gap and limits the ability to design effective new interventions. This research, conducted for Digital Transformations for Health Lab (DTH-Lab) to inform its work on DC4H, set out to identify and evaluate global, regional, and national digital health and civic literacy platforms that promote DC4H. The study examined whether existing platforms met the needs of young people, identified their strengths, and highlighted the gaps that required attention. It also assessed the importance of developing a new platform to address unmet needs and strengthen young people’s digital, health, and civic capacities.

## Method

### Study design

This study adopted a two-phase approach: 1) Platform Mapping and 2) Platform Evaluation. The first phase was the mapping of existing platforms, which identified digital health and civic literacy platforms for young people. The second phase evaluated the identified platforms using walkthrough assessment and document review. A comparative analysis then identified patterns, strengths, and gaps across platforms. Data collection was between June and October 2024, and the findings represent a snapshot of platform features during this period. This study broadly defined digital platforms as tools, resources, and initiatives that support young people’s engagement in DC4H.

### Phase I: Platform mapping

This study used an integrated search approach to identify relevant platforms, combining academic database searches, AI-assisted tools, social media searches, and general web searches. The search queried academic databases (Google Scholar and ResearchGate) using combinations of keywords related to digital citizenship, digital health, civic literacy, and young people. AI-assisted tools (ChatGPT and SciSpace) generated lists of candidate platforms, and targeted searches on social media (X and LinkedIn) and Google identified additional platforms. The result was triangulated, and identified platforms were screened against predefined inclusion and exclusion criteria. The study included platforms if they: (i) addressed digital, health, or civic literacy; (ii) targeted young people; (iii) were publicly accessible for walkthrough assessment; and (iv) provided sufficient content or functionality for evaluation. The study excluded inaccessible platforms that were restricted or required a subscription. The search initially identified 65 platforms. After screening, 38 platforms met the eligibility criteria for inclusion in the final analysis.

### Phase II: Platform evaluation

This study systematically evaluated the identified platforms using walkthrough assessments and document reviews. The walkthrough followed a standardized process in which the researcher accessed each platform, completed registration where required, explored navigation and interface, engaged with core content and available features, and tested interactive components where available [15]. This approach allowed for consistent assessment of platform functionality, usability, and content from a user perspective. The study conducted a document review alongside the walkthrough by examining available platform materials (e.g., descriptions, reports, and supporting content). The researcher assessed each platform using a standardized evaluation framework based on 13 predefined criteria (Table 1), grouped into youth-identified values and features derived from global youth consultations [16] and functional categories aligned with the study objectives.

**Table 1:** Operationalization of evaluation criteria for digital platform assessment.

Domain	Criterion	Operational Definition
Youth-identified values and features	Equity	Ensures fair access across diverse users and contexts, specifically platforms designed with multilingual support, free access, offline functionality, or low-bandwidth design options
	Trustworthy	Ensures that information is accurate, credible, and reliable, assessed through evidence of institutional affiliation, expert involvement, authorship disclosure, or references to validated and peer-reviewed sources
	Humanistic	Focuses on the holistic development of individuals by supporting mental and emotional well-being, assessed through the presence of peer support features, mental health resources, live chat functions, or content addressing empathy and emotional health
	Ethical	Respects user privacy and responsible data practices, assessed through observable indicators such as privacy policies, informed consent mechanisms, data protection statements, or user authentication features
	Inclusive	Ensures accessibility for users with diverse abilities and backgrounds by assessing the availability of alternative formats (e.g., audio/visual), disability-friendly design, and culturally relevant content.
	Everyone interconnected	Promotes communication and interaction among users, as evidenced by forums, discussion boards, or peer-to-peer engagement.
	User-friendly	Ensures ease of navigation and usability through simple interface design, intuitive navigation, and minimal effort to access content.
	Quality personalized services	Provides tailored user experiences, assessed through the availability of personalized recommendations, adaptive content, or individualized support features such as helplines, live chat, peer support, and personalized interaction.
Functional criteria (DTH-Lab objectives)	Knowledge	Supports learning and critical understanding, assessed through the provision of educational content, information resources, or knowledge-based materials.
	Skill	Supports the development of practical abilities through interactive elements such as quizzes, exercises, and skill-building activities.
	Digital literacy	Develops digital competencies and safe technology use, assessed through content addressing digital skills, online safety, or media literacy.
	Health literacy	Supports understanding and use of health information, assessed through the provision of health education, decision-making tools, or health navigation support.
	Civic literacy	Encourages civic awareness and participation, as evidenced by features that enable advocacy, community engagement, and participation in civic activities.

Each criterion in Table 1 was scored as described in the evaluation procedure above.

### Data analysis

Each criterion was operationalized with predefined observable indicators and scored using a binary (Yes/No) approach based on the presence or absence of at least one relevant feature. This approach ensured consistency in assessment and captured the minimum presence of key features across platforms, rather than the degree of performance. The total score for each platform was calculated by summing the number of criteria met, with a maximum score of 13. The study used a 70% threshold (9 out of 13 criteria) to identify platforms that demonstrate stronger alignment with DC4H. Data were analyzed using descriptive and comparative approaches. The distribution of scores across platforms was examined to identify common strengths and gaps. In addition, the number and proportion of platforms meeting each criterion were calculated. The walkthrough assessment was conducted by the researcher (MPH) and reviewed by two public health/digital transformation for health experts at DTH-Lab. To improve reliability, the evaluation framework was discussed before and after data extraction, and discrepancies in scoring were resolved through consensus.

### Ethical considerations

The research consisted exclusively of walkthrough assessments of publicly accessible digital platforms and document review. Therefore, formal ethical approval was not required.

## Results

### Overview of the identified platforms

Through the platform mapping phase, 38 platforms that support young people’s digital, health, and/or civic literacy and skills were identified (S1 Table). These included several educational platforms, such as online courses, webinars, and MOOCs.

The identified platforms varied in their primary focus. Of the 38 platforms reviewed, most (76.3%, n = 29) focused on only one literacy area without addressing the interconnections between digital, health, and civic literacy. Only a minority of platforms demonstrated an integrated approach across these domains. Platforms also differed in their design and functionality. While many provided educational content such as articles, videos, and guides, fewer incorporated interactive or participatory features such as quizzes, discussion forums, or peer-to-peer engagement.

For example, the Digital Citizenship+ Resource Platform (DCPR) provides a diverse collection of learning resources across topics such as privacy, security, and civic engagement, and was designed through youth co-creation through workshops and user testing [17]. The platform includes over 150 educational resources, such as curricula, videos, infographics, and lesson plans, organized across thematic areas including participation, engagement, and well-being. It is available in multiple languages and is designed to be accessible in low-bandwidth settings, supporting use across diverse contexts. The platform primarily provides educational content, with features focused on resource access and information dissemination.

Similarly, the Better Internet for Kids (BIK) platform provides a wide range of educational resources and incorporates youth engagement through advisory groups and participatory initiatives [18]. The platform includes over 1,300 resources and is supported by a network of stakeholders, including policymakers, educators, and civil society organizations. Youth engagement is incorporated through structures such as youth panels and ambassador programmes, which contribute to content development and policy discussions. The platform also includes features such as community forums and tools for engagement with policy-related initiatives.

### Assessment of platform features and comparative patterns

The identified platforms were assessed for their alignment with DC4H principles using 13 predefined criteria. All platforms (100%, n = 38) provided knowledge-based content, reflecting a strong emphasis on information dissemination. However, fewer platforms (44.7%, n = 17) included features that support practical skill development. Among the value-based criteria, most platforms were considered trustworthy (92.1%, n = 35) and user-friendly (84.2%, n = 32), while more than half (55.3%, n = 21) met the ethical criterion. In contrast, equitable access was observed in only 34.2% (n = 13) of platforms, and humanistic features were present in just 26.3% (n = 10). Overall, only seven platforms (18.4%) met at least 70% of the evaluation criteria (Table 2).

**Table 2:** Comparative Assessment of DC4H Platforms Against Evaluation Criteria.

SN	DC4H PLATFORMS	DFHS REPORT YOUTH-IDENTIFIED VALUES AND FEATURES								DTH-LAB OBJECTIVE: FEATURES				
		VALUES					FEATURES			CAPACITY BUILDING		DC4H LITERACY		
		Equitable	Trustworthy	Humanistic	Ethical	Inclusive	QPS	User Friendly	Everyone Interconnected	Knowledge	Skill	Digital	Health	Civic
1	Kolibri	✔	✔		✔	✔		✔		✔	✔	✔	✔	✔
2	Digital Citizenship+ Resource Platform	✔	✔			✔		✔		✔	✔	✔		✔
3	Young Expert Tech for Health Resource Hub		✔							✔		✔	✔	
4	BrainPOP Digital Citizenship		✔		✔			✔		✔	✔	✔		
5	ReachOut		✔	✔	✔	✔	✔	✔	✔	✔			✔	
6	Teen Health and Wellness	✔	✔	✔	✔		✔	✔		✔	✔		✔	
7	Youth Mental Health Project		✔	✔	✔	✔	✔	✔	✔	✔			✔	
8	Mental Health Curriculum and Toolbox		✔			✔		✔		✔			✔	
9	iCivics		✔					✔		✔	✔			✔
10	Teen Line		✔	✔	✔	✔	✔			✔			✔	
11	Learning.com		✔		✔	✔		✔	✔	✔	✔	✔		
12	The Mix	✔	✔	✔	✔		✔	✔	✔	✔			✔	
13	Teen Health Source		✔	✔	✔		✔	✔		✔			✔	
14	Scarleteen		✔	✔	✔		✔	✔		✔			✔	
15	U-Report	✔	✔		✔		✔	✔	✔	✔	✔		✔	✔
16	Webonauts Academy				✔	✔		✔	✔	✔	✔	✔		
17	Digital civics academy		✔				✔		✔	✔	✔	✔		✔
18	Be smart online (Foundation for Social Welfare Service)		✔	✔	✔		✔	✔		✔		✔		
19	Learning for Justice		✔		✔			✔		✔				✔
20	NetSmartz Workshop		✔		✔		✔	✔		✔	✔	✔		
21	Social Media U		✔				✔			✔	✔	✔		
22	Be smart online (Childnet)		✔				✔	✔		✔		✔		
23	Mundo	✔				✔	✔	✔		✔		✔	✔	✔
24	Adolescent & Youth Sexual & Reproductive Health Toolkit		✔		✔		✔	✔	✔	✔	✔		✔	
25	Media Smarts (Canada Centre for Digital Literacy)		✔					✔		✔	✔		✔	
26	Netsafe		✔	✔	✔		✔	✔		✔		✔		
27	My Kialo				✔		✔	✔	✔	✔				✔
28	Civic Tech Field Guide	✔	✔				✔	✔		✔				✔
29	Better Internet for Kids (BIK)	✔	✔		✔	✔	✔			✔	✔	✔		
30	Keep it real online	✔	✔	✔	✔		✔	✔		✔		✔		
31	Media and Information Literacy (MIL)	✔	✔			✔		✔		✔	✔	✔		✔
32	The Civic Center		✔				✔	✔		✔	✔			✔
33	Civics for all		✔					✔		✔				✔
34	Teaching For Democracy Alliance		✔					✔		✔				✔
35	Civics Learning Project		✔		✔			✔		✔	✔			✔
36	Rumie	✔	✔					✔		✔		✔	✔	✔
37	Hesperian Health Guides	✔	✔					✔		✔			✔	
38	All Children Reading: A Grand Challenge for Development (ACR GCD) Library Apps	✔	✔			✔				✔				✔

Source: References [17–54].

**Fig 1:**
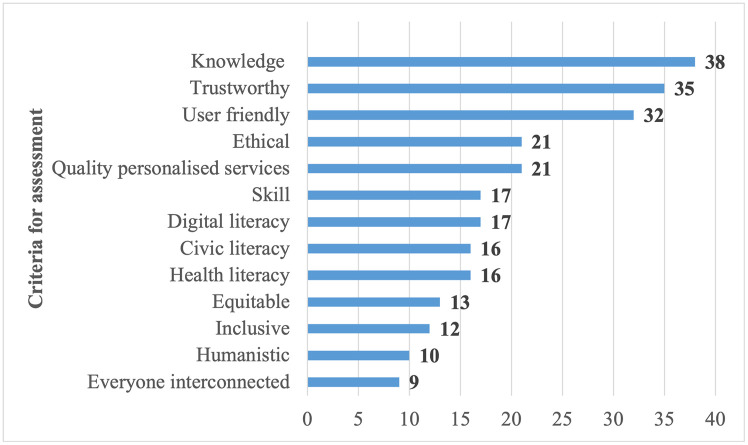
Compliance of evaluated platforms with DC4H evaluation criteria.

A comparison across criteria revealed several patterns in platform design. While knowledge provision was universal, engagement-oriented features such as skill-building, humanistic support, and personalized services were considerably less common. In addition, most platforms focused on a single literacy domain rather than integrating digital, health, and civic literacy within a unified framework (Fig 1).

## Discussion

The findings of this study highlight gaps in how digital platforms currently support DC4H. No platform was found to meet all key values such as equity, trustworthiness, humanistic design, ethics, and inclusivity. This indicates that existing platforms only partially address the needs of young people and suggests the need for more integrated, user-centered approaches.

### Equity and inclusion

Significant gaps were identified, particularly in equity and inclusion. This reflects underlying “competence-based assumptions” and “language centrism” in digital platform design, where users are expected to have high digital literacy, stable internet access, and proficiency in dominant languages. This finding is consistent with the existing literature, which shows that digital health interventions are often designed for users with higher literacy levels and reliable digital access, thereby inadvertently excluding disadvantaged populations. Integrating multilingual functionality potentially improves accessibility for diverse user groups [55,56].

The study also identified other gaps. For example, some platforms do not support users with reading difficulties because most of the core content is text-based. Previous studies have shown that text-heavy digital content can limit accessibility for users with low literacy levels or diverse learning needs, reducing engagement and effectiveness [57,58].

In addition, most platforms assessed depend on internet connectivity, excluding users in low-resource settings where electricity or internet access is unreliable. This highlights structural inequalities in digital access, particularly in low-resource settings. Research on the digital divide has consistently identified limited internet access and infrastructure as key barriers to the equitable use of digital health tools, particularly in low- and middle-income countries [59,60].

### Frameworks for inclusive design

Several established frameworks and principles support the development of inclusive digital platforms and address the gaps identified in this review. An important one is the Sustainable Development Goal (SDG) 4, which promotes equitable access to education at all levels. Other frameworks guiding digital platforms and designs include the Universal Design for Instruction (UDI) and the Web Content Accessibility Guidelines (WCAG). UDI is guided by 7 principles, including equitable use, which ensures that designs accommodate people across a spectrum of abilities and backgrounds [61]. WCAG also provides comprehensive recommendations for creating digital content that serves users with various disabilities, including visual, auditory, cognitive, and motor impairments. When properly implemented, these guidelines benefit not only users with specific accessibility needs but also enhance usability for all users [62]. Additionally, Intersectionality frameworks require developers to consider how multiple, overlapping identities, such as race, gender, socioeconomic status, and disability, can compound barriers to access [63].

### Integration of digital, health, and civic literacy

Most platforms did not integrate digital, health, and civic literacy resources. The majority focused on only one of these areas, with only a few offering comprehensive resources across all three. This fragmented approach likely reflects sector-specific development processes in which digital, health, and civic initiatives are designed independently rather than collaboratively, thereby overlooking their interconnectedness [1].

This interconnectedness is particularly evident in the digital space, which creates opportunities for civic debate, political participation, and public health discourse [64]. Democratic systems with strong institutions, including open expression, fair electoral processes, high public trust, and adherence to legal frameworks, have demonstrated better performance in addressing health crises, advancing universal healthcare coverage, and fostering inclusive dialogue about health policies [1,65].

Evidence from real-world settings reinforces this link. Health systems in democracies tend to enhance political engagement and trust, creating space for open expression on health issues and more responsive policies [66]. This is illustrated by Tunisia’s post-2011 Societal Dialogue, which established inclusive health forums involving citizens, experts, and officials, resulting in democratic health governance and broader policy buy-in [67]. Long-term data from 115 countries (1960–2015) further confirm that democratization causally improves health outcomes, including UHC, by increasing public spending and reducing corruption [68]. WHO guidance on citizen engagement similarly notes that inclusive, evidence-informed policymaking incorporates public preferences, enhancing adherence and equity in health interventions [69].

These findings carry direct implications for platform design. Integrating digital, health, and civic literacy within a single platform is not merely a structural convenience. It reflects how these domains function together in practice. Platforms that treat them in isolation risk undermining users’ capacity to engage meaningfully with health information, participate in civic processes, and navigate the digital environments where both increasingly occur.

### Ethics, trustworthiness, and AI integration

Ethical integrity and trustworthiness are critical values for digital platforms, particularly those incorporating artificial intelligence. Some platforms reviewed here, despite promoting fairness and inclusiveness, did not adequately prioritize security or information quality. This gap is especially concerning for AI-integrated platforms, where the challenge extends beyond access to the reliability and accuracy of the content provided. AI-driven platforms offer comprehensive, on-demand resources that go beyond what traditional platforms provide. However, their ethical implications require careful consideration. AI systems can generate inaccurate information through hallucinations or biases embedded in training data, raising serious concerns about misinformation [70]. The growing literature on digital health and AI consistently highlights these risks, emphasizing issues related to misinformation, bias, and lack of transparency [70].

In health literacy specifically, AI may provide misleading guidance that undermines informed decision-making and autonomy, placing young people at particular risk [71,72]. There are also equity implications: relying on AI without robust verification mechanisms may widen existing gaps, as not all users possess the digital literacy needed to identify and correct AI errors. Addressing these concerns requires stronger governance frameworks that prioritize data protection, transparency, and information verification, including secure data storage, authentication, clear data use policies, and source verification processes, to ensure platforms are both ethical and genuinely trustworthy.

### Humanistic design and user support

Of all the platforms analyzed, only the health literacy platforms included features that were truly compassionate and person-centered, such as hotlines, chatlines, and text-lines that allowed users to ask questions and receive personalized support. This suggests that most platforms prioritize information delivery over user engagement and support, potentially limiting their effectiveness for young users. This pattern has been observed in the broader digital health literature, where information-based approaches are often prioritized over interactive, user-centered design. This gap is particularly consequential for underserved populations, for whom person-centered features are not optional enhancements but essential conditions for meaningful engagement. Without them, platforms may expand access in name while failing to meet users where they actually are [73].

### Youth engagement and platform use

Meaningful engagement, rather than mere access, is what determines whether digital platforms produce lasting impact for young people. Research shows that youth engagement with digital platforms is shaped by factors such as perceived usefulness, ease of use, social influence, digital self-efficacy, and trust [7,74]. These factors align with established technology adoption frameworks such as the Technology Acceptance Model (TAM), which identifies perceived usefulness and perceived ease of use as key determinants of technology adoption. Within the context of DC4H platforms, these determinants suggest that platforms must not only provide credible information but also ensure intuitive design, interactive features, and clear value for users in order to encourage sustained engagement among young people [75].

The DC4H platforms reviewed showed some promising design choices in this regard. Most demonstrated a clear commitment to knowledge sharing and user-friendly design, with features such as intuitive navigation, content filters, and visually appealing landing pages. These elements lower the barrier to initial engagement, and research consistently confirms that young people are more likely to interact with digital tools that feel accessible and easy to use [76]. However, good design alone is insufficient. The limited availability of skill-building resources represents a significant gap, as knowledge acquisition without structured opportunities for application does little to build genuine competency. Incorporating interactive elements such as online campaigns, peer learning, role-play scenarios, and quizzes would enable young people to move from passive consumption to active participation, substantially enhancing the value of DC4H platforms.

The social dimension of engagement is equally important and similarly underdeveloped across the platforms reviewed. Young people thrive in community-oriented environments, and digital spaces that facilitate peer support, collaborative learning, and online forums foster deeper engagement and more sustained knowledge retention [77]. Without deliberate efforts to embed interconnectivity and active skill development, many existing platforms risk remaining informational repositories rather than transformative spaces for youth participation and growth.

### Recommendations for developing new DC4H platforms

The gaps identified in this study point to clear priorities for developing new DC4H platforms. The following recommendations are organized into three sections: platform design, strategic and policy considerations, and future research.

Digital platforms and learning environments for young people should adopt user-centered, inclusive design approaches to better meet the needs of diverse groups. This includes integrating multilingual support, offline functionality, and alternative content formats to improve accessibility, particularly in low-resource settings. Platforms should also incorporate interactive and personalized features, such as peer engagement, feedback mechanisms, and skill-building activities, to enhance usability and sustained engagement. Clear privacy policies, informed consent processes, and credible information sources are essential to ensure that platforms are trustworthy and ethically designed.

At a strategic level, there is a need to support the development of integrated digital platforms that combine digital, health, and civic literacy, rather than addressing these areas in isolation. Policymakers and stakeholders should promote inclusive design standards, data protection, and equitable access. Strengthening partnerships with schools, health promotion organizations, and civic literacy initiatives can enhance the reach and sustainability of platforms. In addition, involving young people through co-creation and advisory processes can ensure that platforms remain relevant and responsive to user needs.

Future research should explore how young people engage with digital platforms, including factors such as usability, trust, and accessibility, to better understand how platforms can support DC4H. Participatory approaches, such as co-design, user testing, and feedback loops, are needed to ensure that platforms align with user needs. Further studies should also examine how to effectively integrate digital, health, and civic literacy within single platforms and assess their long-term impact on knowledge, skills, and behavior change across different contexts.

## Conclusion

These findings provide a critical foundation for developing a new DC4H platform that addresses the identified gaps. Such a platform must be grounded in a deep understanding of the cultural, social, and digital landscapes in which young people live, and designed to be accessible, inclusive, interactive, and relevant across diverse regional contexts. Achieving this requires the active involvement of young people, educators, health professionals, and local organizations throughout the design and development process, ensuring the platform remains responsive to evolving user needs. Ultimately, the goal is not simply to create another information resource, but to build a transformative tool that positions young people as active participants in their own health and civic lives.

### Limitation of the study

One of the primary limitations of this study is the subjectivity inherent in the assessment process. The evaluation relied on predetermined criteria and the researchers’ interpretations, without incorporating direct user or stakeholder feedback. This approach may have introduced bias, as it reflects a limited perspective on the usability, effectiveness, and relevance of DC4H platforms. Although additional researchers reviewed the evaluation, the walkthrough assessment was primarily conducted by a single researcher, which may have influenced the findings.

A second limitation relates to the use of AI-assisted tools and web-based searches for platform identification. While these approaches enabled broad search, they may have introduced selection bias through algorithmic filtering and the visibility of certain platforms over others. In addition, the study was conducted between June and October 2024, and platform features and availability may have evolved since then. As digital platforms are continuously updated, the findings represent a snapshot in time.

To address these limitations, future research should integrate more participatory methods, such as focus group discussions, user interviews, or surveys. These methods would allow for a broader range of perspectives, yielding richer, more diverse insights. Such an approach would help validate the findings, ensuring they better reflect the actual user experience and align with the needs of diverse populations.

## Supporting information

S1 Table Digital, Health, and Civic Literacy Platforms. (XLSX)
