# Supplementary material for: Digital citizenship for health: Mapping and evaluation of existing platforms supporting digital, health, and civic literacy among young people
Source: PLOS Digit Health. 2026 Jul 16;5(7):e0000737. doi: 10.1371/journal.pdig.0000737 (PMC13375011; doi:10.1371/journal.pdig.0000737)
Supplement: Attachment — Submitted filename: Response to reviewers_.docx [file pdig.0000737.s004.docx]

# Author response to reviewers

#### MANUSCRIPT ID: PDIG-D-24-00593

#### MANUSCRIPT TITLE: Digital citizenship for health: Mapping existing platforms for digital, health and civic literacy

Thank you for taking the time to review our manuscript. Your detailed feedback and suggestions have greatly improved the clarity, depth, and overall quality of the work. Please find below a detailed response to all comments from the editors and reviewers.

| REVIEWER 1 COMMENT | AUTHOR RESPONSE | PAGE NUMBER |
| --- | --- | --- |
| Include the ethical consideration in using AI driven tools as a more thorough discussion would be beneficial. | Thank you for the suggestion. We have expanded the discussion to include ethical considerations in the use of AI-driven tools.  **Revised text in manuscript:**  “*Other important values, especially with digital platforms and the integration of artificial intelligence (AI), is ‘ethical’ and ‘trustworthy’. Few platforms were considered not fully ethical or trustworthy. Although they promoted fairness and inclusivity, they did not prioritize security and quality information. An example is the AI-integrated literacy platforms. With AI platforms, the concern extends beyond access to the quality and reliability of the information provided. While AI-driven digital platforms offer comprehensive, on-demand resources that surpass the scope of traditional platforms, their ethical implications must be carefully considered. AI platforms, though versatile, can generate inaccurate information due to 'hallucinations' or biases in training data, raising ethical concerns about misinformation.59 For instance, in health literacy, AI might provide misleading advice that undermines informed decision-making and autonomy, potentially putting young people and other vulnerable groups at risk. Ethically, relying on AI without robust verification could also widen equity gaps, as not everyone has the digital literacy required to identify and correct AI flaws.”* | 23 |
| Also the introduction will benefit from a re-write to improve conciseness and readability to engage the reader throughout. | Thank you for your suggestion. The introduction has been revised for conciseness and readability. These changes are also reflected in the abstract and across the manuscript as well. | 2-3 |
| Another key benefit will be to discuss sustainability of these tools to provide a more comprehensive understanding of their long-term viability. | Thank you for this observation. While a full evaluation of sustainability requires deeper study beyond the scope of this review, we have acknowledged this as an important area for future research and discussion. | - |

| REVIEWER 2 COMMENT | AUTHOR RESPONSE | PAGE NUMBER |
| --- | --- | --- |
| 1. Methodology  Concern: While the study has a clear comprehensive search strategy and evaluation framework, minimal discussion of the set of values and features prioritized by youth during a series of global consultations conducted by the DTH-Lab which were ultimately used as evaluation criteria. | Thank you for this observation. While full details of the consultation are available in the cited reference, we have now added a description to provide more context on how the values and features were developed.  **Revised text in manuscript:**  *“In the global interim report, Building a blueprint for digital first health systems: Findings from global youth consultations, young people from around the world identified a set of core values and features that should inform the design and governance of digital-first health systems. These consultations engaged over 100 young people under the age of 30 from 36 countries through a social media campaign, essay submissions, and virtual discussions with global youth networks. The findings from the report show that systems should not only better predict and prevent physical and mental health challenges in the future but also support young people in promoting and managing their health and well-being today. Assessing resources based on the report's findings ensures that young people’s voices are included in the co-creation of the toolkit.13 The values identified and used for evaluation included ethical, inclusive, trustworthy, equitable, humanistic, everyone connected, quality personalized services and user-friendly.”* | 5 |
| 2. Health Outcomes Integration  Clinical Concern: While the paper evaluates tool features, it lacks further discussion of how these tools might more directly impact actual health outcomes or clinical care.  Suggestions:  Include discussion of evidence-based features that correlate with improved health  Address how these tools might integrate with clinical workflows | Thank you for this important comment. The focus of our study is on health promotion rather than clinical care, so integration with clinical workflows falls outside the scope of this paper. We have, however, expanded the discussion to highlight how the right digital tools can contribute to improved health outcomes by strengthening knowledge, supporting behavior change, reducing exposure to misinformation, and improving access to trusted resources. | 24-26 |
| 3. Clinical Implementation Considerations  Gap Identified: The recommendations section lacks practical guidance for healthcare providers.  Suggestions for Addition:  How clinicians can evaluate and recommend DHC tools  Integration strategies with existing health education protocols  Identifying training needs for healthcare staff | Thank you for this thoughtful comment. Our study focuses on digital citizenship for health from the perspective of young people, with an emphasis on literacy, empowerment, and health promotion rather than clinical practice. For this reason, we did not include detailed recommendations or guidance for healthcare providers. Notwithstanding, we recognise that all stakeholders play a role in ensuring the relevance and sustainability of these tools. This is why we added to the conclusion that developers should include professionals in the development and evaluation process, to ensure the tools are credible, trustworthy, and better connected to broader health systems. | 27-28 |
| Minor Technical Issues and Recommendations  Figure References: A graphical reference on a plot with all 38 tools with X axis and Y axis reflecting # evaluation criteria may be a visually appealing quick reference to see the performance of certain tools over others. Ie. highest performing tool at top right hand corner. Furthermore, an additional graph with evaluation criteria on X axis and # of papers on Y axis will visually summarize most common strengths and gaps among existing tools. | Thank you for this helpful suggestion. A full graphical reference plotting all 38 tools against the evaluation criteria would duplicate the information already presented in the detailed table. To avoid redundancy, we instead added a chart that summarises the most common strengths and gaps across the evaluated tools, as suggested. This provides a concise visual reference while complementing the existing table. | 24 |
| Terminology Consistency: ensure consistent use of either young people, youth and adolescents or clarify specific age group. | Thank you for this observation. We have ensured consistent use of the term “young people” throughout the manuscript. The terms “youth” or “adolescents” are retained only in instances where they are directly tied to the name of a tool, the wording used in a consultation, or the terminology cited in a report. | All |
| Grammar: Civic engagement and advocacy platforms are equally important as it -> they provide a voice for young people to make a difference in several community initiatives while bringing about social change. | Thank you for the observation. This has been corrected. | 3 |
