# Supplementary material for: Digital citizenship for health: Mapping and evaluation of existing platforms supporting digital, health, and civic literacy among young people
Source: PLOS Digit Health. 2026 Jul 16;5(7):e0000737. doi: 10.1371/journal.pdig.0000737 (PMC13375011; doi:10.1371/journal.pdig.0000737)
Supplement: Attachment — Submitted filename: Manuscript Review Report.doc [file pdig.0000737.s005.doc]

**Manuscript Review Report: PDIG-D-24-00593R1**

Manuscript Title: Digital citizenship for health: Mapping existing platforms for digital, health and civic literacy

1. **Overall Evaluation**

This study aims to map and evaluate existing platforms for "Digital Citizenship for Health" (DC4H). The topic is of significant practical importance, particularly in its focus on the needs of young people, aligning well with current trends in the digital health field. The authors have undertaken substantial work in identifying and cataloging resources, collecting data on 38 platforms, and attempting to establish an evaluation framework based on findings from youth consultations.

However, the current version of the manuscript contains serious flaws that prevent it from meeting the standards for publication in terms of scientific rigor and reporting clarity. The most critical issues are concentrated in the Methods section, where the description suffers from structural confusion, omission of key details, and vague definition of criteria, severely undermining the study's credibility and replicability. Furthermore, pervasive inconsistency in terminology and problematic language expression throughout the manuscript further hinder comprehension.

Therefore, I recommend "Major Revision." The authors must undertake a thorough rewrite and clarification of the research methods, coupled with comprehensive language polishing, to enable this potentially valuable study to be fairly assessed and effectively communicated.

2. **Specific Comments (by Section)**

**2.1 Research Methods and Design (Core Area of Criticism)**

This section is the weakest part of the manuscript, plagued by several fundamental problems:

**Illogical Section Structure:**

Stage 1 (Mapping) is titled as mapping existing platforms, yet its description includes conducting a "walkthrough assessment" on all platforms. This conflates the distinct phases of "identification" and "evaluation." Evaluation should reside entirely within Stage 2.

Stage 2 (Assessment of strengths) should detail how the identified platforms were systematically evaluated. Instead, it re-mentions the "walkthrough" concept from Stage 1, abruptly introduces the evaluation criteria (the inclusion/exclusion criteria themselves should be presented in Stage 1), and glosses over the execution of the assessment process (e.g., coding, comparative analysis steps). The entire Methods chapter lacks a clear, linear description of the research workflow.

**Unclear Execution of Evaluation Process and Lack of Reliability:**

Unspecified Executors and Process: The text only states, "A walkthrough assessment was then conducted for each tool." Critical information is missing: who performed it (how many assessors? what were their backgrounds?), how it was performed (was there a standardized evaluation form or protocol?), and how it was documented.

Insufficient Assurance of Reliability and Validity: For assessments involving subjective judgment (e.g., whether something is "humanistic" or "trustworthy"), ensuring inter-rater reliability (e.g., were there multiple independent assessors? How was scoring consistency measured and addressed?) is fundamental to scientific rigor. The manuscript provides no discussion of this, casting serious doubt on the validity of the evaluation results.

**Oversimplified and Poorly Defined Evaluation Criteria:**

Overly Simplistic Dichotomy: The use of a simple "Yes/No" (✔) dichotomy to judge complex constructs like "Equitable," "Inclusive," or "Ethical" is highly problematic and lacks rigor. This simplification fails to capture degrees of performance on these dimensions and is highly susceptible to assessor bias.

Missing Operational Definitions: For instance, what specific features qualify a platform as "Equitable"? Is supporting 5 languages sufficient, or 10? What constitutes a "Humanistic" feature? Is a live chat function alone enough? The lack of clear, observable operational definitions for these criteria renders the evaluation process subjective and its results difficult to interpret or compare.

**Unsound Inclusion and Exclusion Criteria:**

Inclusion Criterion 1 (Grammatical and Logical): "Platforms primarily discussing or exploring or designed for DC4H or related concepts." This sentence has significant grammatical and logical ambiguities, as noted in previous analysis. It fails to clearly define the boundaries of the study's subject.

Inclusion Criterion 3 (Category Mismatch): "Research type: Platforms walkthrough, literature review..." This is confusing. Using "research type" (e.g., literature review) as an inclusion criterion for "platforms" is a conceptual error. Appropriate criteria should be based on "platform type" or "resource format."

Overly Simplistic Exclusion Criterion: The single criterion "Inaccessible sources" completely overlooks important screening dimensions such as a platform's influence (e.g., user base, usage frequency), quality (e.g., evidence-based content), and timeliness (e.g., actively maintained). This calls into question the representativeness and quality of the platform sample.

**2.2 Language, Terminology, and Expression**

**Severe Terminology Inconsistency:** The manuscript interchangeably and indiscriminately uses platforms, tools, resources, and initiatives throughout, especially in the Methods and Results sections. This creates conceptual ambiguity regarding the core subject of study, which is unacceptable in academic writing. A core term must be selected, defined, and used consistently across the entire text.

**Awkward and Unprofessional Language Expression:** As noted in the overall evaluation, the manuscript contains numerous sentences that are verbose, poorly structured, misuse prepositions, or have unclear pronoun references. This not only impedes readability but may also obscure or distort the authors' intended meaning. The authors are strongly advised to seek professional language editing services.

**Occasionally Overstated Conclusions:** For example, the statement in the Abstract, "Capacity building should also be prioritized over information sharing," draws a broad conclusion based on this study's evaluation. Stating this so definitively here seems premature; it would be more appropriately positioned in the Discussion section as a cautious implication derived from the findings.

**2.3 Results and Discussion**

**Presentation of Results:** Table 1 contains useful information but requires formatting optimization for readability. The choice of a simple bar chart (Figure 1) to display compliance with 13 criteria may not be the most effective visualization. Alternatives like a heatmap could better illustrate patterns and differences across platforms.

**Discussion Section:**

Repetition of Introduction: The opening of the Discussion should avoid restating the Results and instead move directly into interpreting the results, comparing them with prior literature, and exploring their implications.

Recommendations Require Depth: The current recommendations are somewhat generic. They could be structured more effectively, for instance, into "Technical recommendations for platform developers," "Strategic recommendations for policymakers," and "Academic recommendations for future research," to enhance their specificity and actionable nature.

3. **Summary and Recommendations for Revision**

**3.1 Decision: Major Revision**

The foundational work of this study has value, but the current manuscript has major flaws in methodological rigor, reporting clarity, and language quality. The reliability of the findings cannot be judged until these issues are resolved.

**3.2 Core Recommendations for the Authors:**

**3.2.1Completely Rewrite the "Methods" Section:**

Clearly Separate Phases: Distinctly separate the "Identification/Mapping" and "Evaluation/Analysis" phases.

Detail the Evaluation Process: Specify assessor details (number, background, training), the specific tool used for evaluation (e.g., evaluation form/protocol), and the assessment procedure (independent evaluation, discussion, mechanism for resolving discrepancies).

Define Evaluation Criteria: Provide clear, operational definitions and graded examples for each evaluation criterion (e.g., define "Inclusive" as High/Medium/Low with specific features for each level). Abandon the simplistic binary (Yes/No) approach.

Reconstruct Inclusion/Exclusion Criteria: Use clear, unambiguous language to establish criteria based on platform attributes (e.g., primary function, target audience, medium) and basic quality thresholds (e.g., publicly accessible, primary language, updated within the last X years).

**3.2.2Standardize and Define Terminology:** Choose and consistently use one primary term (e.g., digital resources) throughout the manuscript, providing a definition upon its first use. Other related terms can be used subsequently as sub-categories.

**3.2.3Undertake Comprehensive Language Polishing:** It is strongly recommended that the manuscript be polished by a native English speaker or professional academic editor, focusing on sentence structure, precise word choice, and formal academic tone.

**3.2.4Deepen the Discussion:** Reduce repetition of results, increase explanation for the findings, engage in dialogue with relevant theories, and make recommendations more specific and structured.

Only through these fundamental revisions can the important contribution of this study be presented clearly and credibly. I look forward to reviewing a revised manuscript.
