# Supplementary material for: Digital citizenship for health: Mapping and evaluation of existing platforms supporting digital, health, and civic literacy among young people
Source: PLOS Digit Health. 2026 Jul 16;5(7):e0000737. doi: 10.1371/journal.pdig.0000737 (PMC13375011; doi:10.1371/journal.pdig.0000737)
Supplement: Attachment — Submitted filename: Response_to_reviewers__auresp_2.docx [file pdig.0000737.s006.docx]

# Author response to reviewers

**MANUSCRIPT ID: PDIG-D-24-00593R1**

**MANUSCRIPT TITLE: Digital citizenship for health: Mapping and evaluation of existing platforms supporting digital, health, and civic literacy among young people**

Thank you for taking the time to review our manuscript. Your detailed feedback and suggestions have greatly improved the clarity, depth, and overall quality of the work. Please find below a detailed response to all comments from the editors and reviewers.

|  |  | Page |
| --- | --- | --- |
| **Editors Comments** | | |
| 1.Methods section:  Clarify the overall study workflow by clearly distinguishing the platform identification (mapping) phase from the evaluation (walkthrough) phase. Please also provide a concise description of who conducted the walkthrough assessments and how the evaluations were performed. | **Method**  The Methods section has been revised to clearly distinguish between the platform identification (mapping) phase and the evaluation (walkthrough) phase. The workflow is now presented as a two-phase approach, explicitly outlining the identification process followed by the evaluation.  Additional details have been included describing the walkthrough procedure, including how platforms were accessed, explored, and assessed. The roles of the assessors are now clearly specified, including the involvement of additional researchers in reviewing the evaluation to improve reliability. | 5 |
| 2.Evaluation criteria:  Provide brief operational clarifications for the evaluation criteria (e.g., ethical, trustworthy, humanistic) and clarify that the binary indicators reflect the presence or absence of predefined observable features. | **Method**  The evaluation criteria have been revised and presented in a structured table (Table 1), with each criterion clearly defined using operational definitions. Each definition now combines conceptual meaning with observable indicators used during assessment.  The scoring approach has been clarified. | 6 |
| 3.Terminology and language:  Ensure consistent use of key terms throughout the manuscript and improve language clarity and academic tone. Professional language polishing is recommended. | **Whole Manuscript**  Thank you for this valuable comment. The manuscript has been carefully revised to improve clarity, readability, and academic tone. Language and grammar have been edited throughout, and professional editing tools were used to enhance sentence structure and consistency. | 7 |
| 4.Discussion and presentation:  Reduce repetition of results in the Discussion and sharpen the interpretation and implications of the findings. Minor formatting improvements to tables and figures may also enhance readability. | **Discussion**  Thank you for this comment. The Discussion section has been revised to reduce repetition of results and to strengthen the interpretation and implications of the findings. In addition, tables and figures have been reformatted to improve clarity and readability. | 12 |
| **Reviewer 3** | | |
| 1. This assessment relies heavily on the author's interpretation, as noted in the Discussion section. Without a second reviewer or a clear protocol, the data may be influenced by confirmation bias. | **Method**  The Methods section has been revised to clarify the evaluation process and reduce concerns regarding subjectivity. The walkthrough assessment was conducted by the lead researcher and reviewed by two additional researchers with expertise in public health and digital transformation for health.  To improve reliability, the evaluation framework was discussed before and after data extraction, and discrepancies in scoring were resolved through consensus. | 7 |
| 2. The paper identifies "Ethical" and "Trustworthy" as important criteria but does not clearly define these terms. The discussion about why some platforms do not meet these criteria lacks detailed information about essential data protection methods, such as encryption and decentralized storage, which are crucial for health-related platforms. | **Method**  Operational definitions for all evaluation criteria, including “Ethical” and “Trustworthy,” have been clarified and presented in Table 1. These definitions specify observable indicators such as privacy policies, consent mechanisms, and evidence of institutional affiliation or validated sources. | 6 |
| 3. While the paper focuses on "young people," it does not discuss what influences this age group to adopt or reject digital tools. Important factors like social influence and digital self-efficacy should be examined. | **Discussion**  Thank you for this valuable comment. The discussion has been expanded to include in the discussion on factors influencing engagement and use of digital platforms  **Theme:** Youth engagement and platform use | 16 |
| 4. Clearly explain the scoring system for the 13 criteria. Detail what a "pass" or "fail" means for subjective criteria like "Humanistic." | **Method**  The scoring system has been clarified in the Methods section. Each criterion was assessed using a binary system (Yes/No), where “Yes” indicates the presence of at least one predefined observable feature. | 6 |
| 5. If possible, include a second researcher to validate a sample of the platforms to assess agreement. If this is not possible, add a limitations section explaining how bias was reduced. | **Method**  Details of the evaluation process and reliability measures have been expanded. | 7 |
| 6. In the Introduction (when discussing young people's digital habits) or in the Discussion (when examining gaps in user engagement. It is suggested to refer from the paper: “Mobile banking adoption: A multi-factorial study on social influence, compatibility, digital self-efficacy, and perceived cost among Generation Z consumers in the United States “. The paper emphasizes "young people" (Generation Z) but lacks research on what drives this group’s behavior. This study examines the adoption of sensitive digital services, such as banking, among Generation Z, which is comparable to the sensitivity of health data. Including this citation will support your claims about why current platforms may not effectively engage youth. | **Discussion**  Thank you for this helpful suggestion. While the suggested study offers valuable insights into Generation Z technology adoption, it focuses on financial services in a high-income country context, which differs methodologically and contextually from the exploratory platform evaluation conducted in this study. The discussion of youth engagement has instead been grounded in literature more directly relevant to digital health and civic literacy platforms. Nevertheless, factors such as social influence and digital self-efficacy are relevant to understanding youth engagement with digital health platforms more broadly, and this has been noted as a direction for future research | 16 |
| 7. The manuscript should enhance its discussion of Generation Z's behavior, security and privacy methods in digital health, and what "trustworthy" systems look like. Expand on what "Trustworthy" and "Ethical" mean for platforms. Currently, the paper states that they lack security but does not explain what adequate security measures are for health platforms. Discuss modern technologies, such as blockchain and advanced encryption, which are setting standards for protecting digital health data. | **Method**  The focus of this study was on observable platform-level features accessible through walkthrough assessment, rather than technical back-end infrastructure. Therefore, the evaluation of certain criteria was limited to publicly visible indicators. A detailed technical security audit (e.g., encryption standards, blockchain, decentralized storage) falls beyond the methodological scope of this exploratory platform review. | 6 |
| 8. The paper claims that tools for youth should be "user-friendly." Incorporate research that analyzes how Generation Z adopts technology. Address factors such as social influence and perceived costs or ease of use to explain why current platforms may struggle to engage them. | **Method/Discussion**  Thank you for this suggestion. The discussion has been expanded to include factors influencing young people's engagement with digital platforms. Platforms requiring subscription or payment before access were also excluded from the study. | 5/16 |
| 9. Provide a clear, step-by-step explanation of the walkthrough process. For instance, say that "the researcher registered as a user and attempted to access X content." | **Method**  Thank you. This has been included in the manuscript. | 5 |
| **Reviewer 4** | | |
| 1、The tables on pages 12-22 are truncated in multiple places, preferably in the same paragraph on the same page, otherwise it will affect the integrity of the information. Suggest ensuring that all columns are fully displayed or consider uploading the table as supplementary material. | **Supplementary file**  Thank you for this suggestion. The table has been revised and moved to the supplementary materials. A reference to the supplementary table has been added in the main text. | - |
| 2、Some references lack DOI or access dates. Suggest adopting the citation format required by the journal and supplementing missing DOIs or stable links. | **References**  Thank you for this comment. The reference list has been revised to ensure consistency with journal guidelines. Missing DOIs, URLs, and access dates have been added where applicable. | - |
| 3、The main text does not specify the executor, duration, whether multiple people evaluate or verify the research method, or how to ensure consistency. Suggest providing additional information on the evaluator's background, evaluation process, recording methods, and how to ensure reliability and validity. | **Method**  Thank you for this comment. Additional details have been included in the Methods section to clarify the evaluation process. | 7 |
| 4、How many tools were initially found in the search? How to filter step by step according to the ranking to the last 38, it is recommended to briefly explain to enhance credibility. Clarify the screening process and consider using PRISMA flowchart to assist in explanation. | **Method**  A PRISMA flowchart was considered but given the exploratory and non-systematic nature of the search, a narrative description of the screening process was retained instead | 5 |
| 5、Only use in the text“ ✔” Indicating compliance with standards, the number or proportion of tools that meet each standard has not been counted. Suggest adding a concluding paragraph or data to explain the compliance rate of each standard. | **Result**  Thank you for this comment. The Results section has been revised to include the number and proportion of platforms meeting each evaluation criterion, providing a clearer summary of compliance across platforms. | 8 |
| 6、Suggest explaining in the limitations section the potential biases that AI retrieval may bring and how to reduce them. | **Limitation**  The potential limitations of AI-assisted identification have also been acknowledged in the limitations section. | 18 |
| 7、In the text, "multilingual support" is considered "equitable" and "accessible format" is considered "inclusive", but it is not clearly defined in the methods section. | **Method**  All evaluation criteria has been included with clear definitions and observable indicators. | 6 |
| 8、 It is recommended to use "tools/resources", "platforms", and "initiatives" interchangeably in the text. | **Method**  Thank you for this suggestion. To improve clarity and consistency, the manuscript has been revised to use “digital platforms” as the primary term throughout, with related terms (e.g., tools, resources, initiatives) used only where appropriate. | 5 |
| 9、During the discussion, it was mentioned that 'most tools lack multilingual support', but the results section did not explicitly present data support. Suggest citing specific values | **Method**  Thank you. The operational definition now provide clarification to how the platforms were evaluated. With certain features, like multilingual support, access under “equity” | 6 |
| 10、Suggest adding a 'Digital Health Citizenship framework diagram' to visually display the intersection of digital, health, and civic literacy. If not applicable, please ignore | Thank you for this suggestion. This was not applicable |  |
| 11、I think most of the tools mentioned in the article are from Europe and America. Should we discuss the problem and countermeasures of the lack of tools in the global South. | **Discussion**  Thank you for this comment. The search and identification of platforms was conducted globally, and the platforms identified, while largely developed in certain regions, are available for global use. Although this study focused on evaluating existing platforms against predefined criteria, the lack of platforms originating from the Global South is an important gap that warrants further investigation and has been noted as a direction for future research | 18 |
| 13、The abstract points out that "most tools lack multilingual support and alternative formats, excluding users with disabilities or limited digital access". This important discovery seems to be expressed as a universal accessibility issue. Please clarify: within your research framework, should this be specifically understood as' failure to meet the specific needs of a diverse subset of young people within the group, such as non-native speakers, disabled youth, and youth from resource poor areas'? | **Abstract**  Thank you for this comment. The statement has been clarified to specify that accessibility challenges affect subgroups of young people, including those with disabilities, limited digital access, and language barriers. | 2 |
| 14、In the introduction, you pointed out that the framework aims to empower marginalized groups. However, categorizing 'young people' directly as a 'marginalized group' may be too vague. Do you mean that young people are generally marginalized in the digital health ecosystem, or do you specifically refer to specific subgroups that are disadvantaged due to digital divide, socioeconomic status, disability, or geographical factors? | **Introduction**  Thank you for this comment. Although the term “marginalized” has been removed for clarity, it meant young people who are disadvantaged due certain factors. | 3-4 |
| 15、In the introduction, As mentioned earlier, most young people lack the ability to critically evaluate information and protect privacy. The fifth paragraph immediately begins to discuss the necessity of meaningful co creation and participation. There is a logical leap in between. Is "participation in co creation" itself a key capacity building or empowerment process within the DHC framework for young people who currently lack the necessary skills? | **Introduction**  Thank you for the observation. Participation in co-creation can also serve as a capacity-building process. Actively engaging in the design and development can help young people develop critical thinking. It also allows the design to meet young people where they are at and accommodate for diverse needs within a platform. | 3-4 |
| 16、When discussing digital tools as a solution, the original text uses "anti bullying tools" as an example and refers to it as "enhancing young people's ability to resist harassment". This is different in nature from the "empowering" abilities emphasized earlier, such as critical thinking, active participation, and co creation. So, in the DHC framework, what is the relationship between protective and reactive "response capabilities" and proactive "empowerment capabilities"? Do they belong to different levels or stages of literacy? | **Introduction**  Thank you for this important comment. In the DHC framework, protective and reactive response capabilities and proactive empowerment capabilities are both relevant across the three literacy forms and do not represent different levels or stages of literacy. Rather, they describe different dimensions of digital competence. Response capabilities focus on managing risks and ensuring safe engagement in digital environments, whereas empowerment capabilities emphasize critical thinking, active participation, and co-creation. While response skills can be considered foundational because they create the conditions necessary for safe participation, empowerment reflects a more transformative expression of digital agency. | 4 |
| 17、The discussion section effectively summarizes the "what are the gaps", but there is insufficient exploration of "why do these gaps exist" and "what do these gaps mean". For example, does the widespread lack of multilingual and alternative formats in tools reveal the "competence-based assumption" and "language centrism" in current digital health development? Does the current situation of "fragmentation" reflect the failure of interdisciplinary collaboration, or is it the neglect of comprehensive projects by funding mechanisms? Suggest conducting a thorough analysis of these driving factors. | **Discussion**  Thank you for this comment. The Discussion has been expanded to explore potential reasons for the identified gaps. |  |
| **Reviewer 5** | | |
| Illogical Section Structure:  Stage 1 (Mapping) is titled as mapping existing platforms, yet its description includes conducting a "walkthrough assessment" on all platforms. This conflates the distinct phases of "identification" and "evaluation." Evaluation should reside entirely within Stage 2.  Stage 2 (Assessment of strengths) should detail how the identified platforms were systematically evaluated. Instead, it re-mentions the "walkthrough" concept from Stage 1, abruptly introduces the evaluation criteria (the inclusion/exclusion criteria themselves should be presented in Stage 1), and glosses over the execution of the assessment process (e.g., coding, comparative analysis steps). The entire Methods chapter lacks a clear, linear description of the research workflow. | **Method**  Thank you for your suggestion. The Methods section has been restructured to clearly separate the identification (mapping) phase and the evaluation phase. | 5 |
| Unclear Execution of Evaluation Process and Lack of Reliability:  Unspecified Executors and Process: The text only states, "A walkthrough assessment was then conducted for each tool." Critical information is missing: who performed it (how many assessors? what were their backgrounds?), how it was performed (was there a standardized evaluation form or protocol?), and how it was documented.  Insufficient Assurance of Reliability and Validity: For assessments involving subjective judgment (e.g., whether something is "humanistic" or "trustworthy"), ensuring inter-rater reliability (e.g., were there multiple independent assessors? How was scoring consistency measured and addressed?) is fundamental to scientific rigor. The manuscript provides no discussion of this, casting serious doubt on the validity of the evaluation results. | **Method**  Additional details have been included describing the walkthrough procedure, including how platforms were accessed, explored, and assessed. The use of a structured evaluation framework and standardized assessment approach has also been clarified.  The Methods section now includes details on how reliability was addressed. The evaluation was reviewed by two additional researchers, and discrepancies were resolved through consensus. This process was implemented to improve consistency and reduce subjectivity. | 5-7 |
| Oversimplified and Poorly Defined Evaluation Criteria:  Overly Simplistic Dichotomy: The use of a simple "Yes/No" (✔) dichotomy to judge complex constructs like "Equitable," "Inclusive," or "Ethical" is highly problematic and lacks rigor. This simplification fails to capture degrees of performance on these dimensions and is highly susceptible to assessor bias.  Missing Operational Definitions: For instance, what specific features qualify a platform as "Equitable"? Is supporting 5 languages sufficient, or 10? What constitutes a "Humanistic" feature? Is a live chat function alone enough? The lack of clear, observable operational definitions for these criteria renders the evaluation process subjective and its results difficult to interpret or compare. | **Method**  All evaluation criteria have been clearly defined in Table 1 using operational definitions that combine conceptual meaning with observable indicators. This ensures transparency and reduces ambiguity in the evaluation process.  The use of a binary scoring system has been justified in the Methods section. The approach was used to ensure consistency across platforms and to allow for comparative analysis. The scoring reflects the presence of minimum criteria rather than the degree of performance, which aligns with the exploratory nature of the study. | 6-7 |
| Unsound Inclusion and Exclusion Criteria:  Inclusion Criterion 1 (Grammatical and Logical): "Platforms primarily discussing or exploring or designed for DC4H or related concepts." This sentence has significant grammatical and logical ambiguities, as noted in previous analysis. It fails to clearly define the boundaries of the study's subject.  Inclusion Criterion 3 (Category Mismatch): "Research type: Platforms walkthrough, literature review..." This is confusing. Using "research type" (e.g., literature review) as an inclusion criterion for "platforms" is a conceptual error. Appropriate criteria should be based on "platform type" or "resource format."  Overly Simplistic Exclusion Criterion: The single criterion "Inaccessible sources" completely overlooks important screening dimensions such as a platform's influence (e.g., user base, usage frequency), quality (e.g., evidence-based content), and timeliness (e.g., actively maintained). This calls into question the representativeness and quality of the platform sample. | **Method**  The inclusion and exclusion criteria have been revised to improve clarity, consistency, and methodological rigor. The previous ambiguous phrasing has been removed, and criteria are now clearly defined based on platform characteristics rather than research type.  Specifically, inclusion criteria now focus on platforms that: (i) address digital, health, or civic literacy; (ii) target young people; (iii) are publicly accessible for walkthrough assessment; and (iv) provide sufficient content or functionality for evaluation. While excluding payment based platforms | 5 |
| Language, Terminology, and Expression  Severe Terminology Inconsistency: The manuscript interchangeably and indiscriminately uses platforms, tools, resources, and initiatives throughout, especially in the Methods and Results sections. This creates conceptual ambiguity regarding the core subject of study, which is unacceptable in academic writing. A core term must be selected, defined, and used consistently across the entire text.  Awkward and Unprofessional Language Expression: As noted in the overall evaluation, the manuscript contains numerous sentences that are verbose, poorly structured, misuse prepositions, or have unclear pronoun references. This not only impedes readability but may also obscure or distort the authors' intended meaning. The authors are strongly advised to seek professional language editing services.  Occasionally Overstated Conclusions: For example, the statement in the Abstract, "Capacity building should also be prioritized over information sharing," draws a broad conclusion based on this study's evaluation. Stating this so definitively here seems premature; it would be more appropriately positioned in the Discussion section as a cautious implication derived from the findings. | **Whole Manuscript**  Thank you for these important comments. The manuscript has been thoroughly revised to address issues related to terminology, language, and interpretation of findings.  First, to improve conceptual clarity, terminology has been standardized throughout the manuscript. The term “digital platforms” is now used consistently as the primary term, with related terms (e.g., tools, resources, initiatives) used only where appropriate and clearly defined at first use.  Second, the manuscript has undergone comprehensive language editing to improve clarity, readability, and academic tone. Sentence structure, grammar, and phrasing have been refined throughout, and professional editing tools were used to ensure consistency and coherence.  Repetition or overstating of conclusions have been removed where necessary |  |
| Results and Discussion  Presentation of Results: Table 1 contains useful information but requires formatting optimization for readability. The choice of a simple bar chart (Figure 1) to display compliance with 13 criteria may not be the most effective visualization. Alternatives like a heatmap could better illustrate patterns and differences across platforms. | **Result**  Thank you for this helpful suggestion. The use of heatmap was carefully considered to visualize patterns across platforms. However, given the exploratory nature of the study and the primary aim of illustrating the overall distribution of compliance across the 13 criteria, the bar chart was retained (Figure 1) as it provides a clear and straightforward summary of frequency and proportion. To improve readability, Table 1 has been moved to the supplementary material | 10-11 |
| Discussion Section:  Repetition of Introduction: The opening of the Discussion should avoid restating the Results and instead move directly into interpreting the results, comparing them with prior literature, and exploring their implications. | **Discussion**  Thank you for this helpful comment. The opening of the Discussion has been revised to focus on the interpretation and implications of the findings rather than restating the results. | 14 |
| Recommendations Require Depth: The current recommendations are somewhat generic. They could be structured more effectively, for instance, into "Technical recommendations for platform developers," "Strategic recommendations for policymakers," and "Academic recommendations for future research," to enhance their specificity and actionable nature. | **Recommendation**  Thank you for this valuable suggestion. The recommendations section has been revised to improve clarity, specificity, and actionable detail. The recommendations have been reorganized into three focused areas, as recommended by another reviewer, addressing platform development, strategic and policy considerations, and future research. | 16 |
| Standardize and Define Terminology: Choose and consistently use one primary term (e.g., digital resources) throughout the manuscript, providing a definition upon its first use. Other related terms can be used subsequently as sub-categories. | **Method**  Thank you for this comment. The manuscript has been revised, and it defined “digital platforms” as the primary term throughout. Related terms (e.g., tools, resources, initiatives) are used where appropriate as sub-categories. | 5 |
