# Supplementary material for: Digital citizenship for health: Mapping and evaluation of existing platforms supporting digital, health, and civic literacy among young people
Source: PLOS Digit Health. 2026 Jul 16;5(7):e0000737. doi: 10.1371/journal.pdig.0000737 (PMC13375011; doi:10.1371/journal.pdig.0000737)
Supplement: Peer review history

# Peer review: Decision Letter 0

**DOI:** 10.1371/journal.pdig.0000737.r001

**Authors:** Aline Lutz de Araujo

8 Sep 2025

PDIG-D-24-00593Digital health citizenship: Mapping existing tools for digital, health and civic literacyPLOS Digital Health Dear Dr. Ogundipe, Thank you for submitting your manuscript to PLOS Digital Health. The reviewers suggested minor revisions. Therefore, we invite you to submit a revised version of the manuscript that addresses the points raised during the review process. Please submit your revised manuscript within 30 days Oct 08 2025 11:59PM. If you will need more time than this to complete your revisions, please reply to this message or contact the journal office at digitalhealth@plos.org. When you're ready to submit your revision, log on to https://www.editorialmanager.com/pdig/ and select the 'Submissions Needing Revision' folder to locate your manuscript file. Please include the following items when submitting your revised manuscript:* A rebuttal letter that responds to each point raised by the editor and reviewer(s). You should upload this letter as a separate file labeled 'Response to Reviewers'. This file does not need to include responses to any formatting updates and technical items listed in the 'Journal Requirements' section below.* A marked-up copy of your manuscript that highlights changes made to the original version. You should upload this as a separate file labeled 'Revised Manuscript with Track Changes'.* An unmarked version of your revised paper without tracked changes. You should upload this as a separate file labeled 'Manuscript'. If you would like to make changes to your financial disclosure, competing interests statement, or data availability statement, please make these updates within the submission form at the time of resubmission. Guidelines for resubmitting your figure files are available below the reviewer comments at the end of this letter. We look forward to receiving your revised manuscript. Kind regards, Aline Lutz de AraujoSection EditorPLOS Digital Health Leo Anthony CeliEditor-in-ChiefPLOS Digital Healthorcid.org/0000-0001-6712-6626 **Journal Requirements:**

1. Please ensure that your Ethics Statement is available in its entirety at the beginning of your Methods section, under a subheading 'Ethics Statement'.

2. We ask that a manuscript source file is provided at Revision. Please upload your manuscript file as a .doc, .docx, .rtf or .tex.

3. Please provide an Author Summary. This should appear in your manuscript between the Abstract (if applicable) and the Introduction, and should be 150–200 words long. The aim should be to make your findings accessible to a wide audience that includes both scientists and non-scientists. Sample summaries can be found on our website under Submission Guidelines:

https://journals.plos.org/digitalhealth/s/submission-guidelines#loc-parts-of-a-submission

4. Please provide a complete Data Availability Statement in the submission form, ensuring you include all necessary access information or a reason for why you are unable to make your data freely accessible. If your research concerns only data provided within your submission, please write "All data are in the manuscript and/or supporting information files" as your Data Availability Statement.

If the reviewer comments include a recommendation to cite specific previously published works, please review and evaluate these publications to determine whether they are relevant and should be cited. There is no requirement to cite these works unless the editor has indicated otherwise. **Additional Editor Comments (if provided):**  Reviewer #1:

Reviewer #2:[Note: HTML markup is below. Please do not edit.]**Reviewers' Comments:**  Reviewer's Responses to Questions

**Comments to the Author**

1. Does this manuscript meet PLOS Digital Health’s publication criteria? Is the manuscript technically sound, and do the data support the conclusions? The manuscript must describe methodologically and ethically rigorous research with conclusions that are appropriately drawn based on the data presented.

Reviewer #1: Yes

Reviewer #2: Yes

**********

2. Has the statistical analysis been performed appropriately and rigorously?

Reviewer #1: Yes

Reviewer #2: Yes

**********

3. Have the authors made all data underlying the findings in their manuscript fully available (please refer to the Data Availability Statement at the start of the manuscript PDF file)?

The PLOS Data policy requires authors to make all data underlying the findings described in their manuscript fully available without restriction, with rare exception. The data should be provided as part of the manuscript or its supporting information, or deposited to a public repository. For example, in addition to summary statistics, the data points behind means, medians and variance measures should be available. If there are restrictions on publicly sharing data—e.g. participant privacy or use of data from a third party—those must be specified.

Reviewer #1: Yes

Reviewer #2: Yes

**********

4. Is the manuscript presented in an intelligible fashion and written in standard English?

PLOS Digital Health does not copyedit accepted manuscripts, so the language in submitted articles must be clear, correct, and unambiguous. Any typographical or grammatical errors should be corrected at revision, so please note any specific errors here.

Reviewer #1: Yes

Reviewer #2: Yes

**********

5. Review Comments to the Author

Please use the space provided to explain your answers to the questions above. You may also include additional comments for the author, including concerns about dual publication, research ethics, or publication ethics. (Please upload your review as an attachment if it exceeds 20,000 characters)

Reviewer #1: The noted strengths of the study includes its timeliness and relevance as an aid for young people to navigate these spaces effectively. The objectives are clear, with gaps adequately identified offering practical recommendations. Aspects to address include the ethical consideration in using AI driven tools as a more thorough discussion would be beneficial. Also the introduction will benefit from a re-write to improve conciseness and readability to engage the reader throughout. Another key benefit will be to discuss sustainability of these tools to provide a more comprehensive understanding of their long-term viability.

Reviewer #2: Overall Assessment

This manuscript presents a timely and relevant analysis of digital health citizenship (DHC) tools designed for young people particularly accelerated with the increasing utilization of AI in healthcare. If we hope to optimize the positive impact of rapidly advancing technologies in healthcare to bridge gaps towards universal healthcare, the importance of digital health citizenship among youth is of particular relevance. While this study focused primarily on evaluating tools impacting youth and adolescents in the realm of digital health citizenship, my experience as a physician and educator has taught me that similar gaps exist in the realm of digital tools promoting AI literacy among clinicians in practice. Clear identification of gaps in accessibility and skills building among existing digital health tools for youth lay the groundwork for more impactful DHC toolkits.

Major Strengths

1. Clinical Relevance and Timeliness

The paper addresses a critical gap in our understanding of digital health tools for youth—a population that is both highly digital-native yet often struggles with health literacy.

2. Comprehensive Evaluation Framework

Including criteria identified young people such as (Equitable, Trustworthy, Humanistic, Ethical, and Inclusive) provides a robust framework that aligns well with medical ethics principles and health equity considerations that we prioritize in clinical practice. Practicing inclusivity in the methodology serves to strengthen overall recommendations towards a DHC toolkit.

3. Clear Comprehensive Search Strategy

The study employed a clear two-stage research design, beginning with a landscape analysis and followed by a strength assessment. It utilized a comprehensive search strategy that incorporated diverse sources, including academic databases, AI tools, and social media. A systematic walkthrough methodology was used to enable practical and consistent evaluation with well-defined inclusion and exclusion criteria.

Areas Requiring Clarification

1. Methodology

Concern: While the study has a clear comprehensive search strategy and evaluation framework, minimal discussion of the set of values and features prioritized by youth during a series of global consultations conducted by the DTH-Lab which were ultimately used as evaluation criteria.

2. Health Outcomes Integration

Clinical Concern: While the paper evaluates tool features, it lacks further discussion of how these tools might more directly impact actual health outcomes or clinical care.

Suggestions:

Include discussion of evidence-based features that correlate with improved health

Address how these tools might integrate with clinical workflows

3. Clinical Implementation Considerations

Gap Identified: The recommendations section lacks practical guidance for healthcare providers.

Suggestions for Addition:

How clinicians can evaluate and recommend DHC tools

Integration strategies with existing health education protocols

Identifying training needs for healthcare staff

Minor Technical Issues and Recommendations

Figure References: A graphical reference on a plot with all 38 tools with X axis and Y axis reflecting # evaluation criteria may be a visually appealing quick reference to see the performance of certain tools over others. Ie. highest performing tool at top right hand corner. Furthermore, an additional graph with evaluation criteria on X axis and # of papers on Y axis will visually summarize most common strengths and gaps among existing tools.

Terminology Consistency: ensure consistent use of either young people, youth and adolescents or clarify specific age group.

Grammar: Civic engagement and advocacy platforms are equally important as it -> they provide a voice for young people to make a difference in several community initiatives while bringing about social change.

Conclusion

This manuscript addresses an important topic relevant to both digital health and clinical practice in a landscape of rapidly advancing technologies with AI in healthcare. The systematic assessment approach is clear, and the findings have practical implications for healthcare providers working with young patients. The incorporation of young people’s perspectives in the evaluation criteria is both ethical and increases the likelihood that digital health tools will be more responsive to the evolving needs of youth.

The paper has the potential to significantly contribute to the literature and development of digital health tools for youth, particularly if the authors enhance the clinical relevance discussion and clinical integration recommendations.

Final Recommendation: Accept with Minor Revisions

**********

6. PLOS authors have the option to publish the peer review history of their article (what does this mean?). If published, this will include your full peer review and any attached files.

**Do you want your identity to be public for this peer review?**   If you choose “no”, your identity will remain anonymous but your review may still be made public.

For information about this choice, including consent withdrawal, please see our Privacy Policy.

Reviewer #1: **Yes:**  Nana Ofori Adomako

Reviewer #2: **Yes:**  Mena Ramos

**********

[NOTE: If reviewer comments were submitted as an attachment file, they will be attached to this email and accessible via the submission site. Please log into your account, locate the manuscript record, and check for the action link "View Attachments". If this link does not appear, there are no attachment files.]**Figure resubmission:**   While revising your submission, we strongly recommend that you use PLOS’s NAAS tool (https://ngplosjournals.pagemajik.ai/artanalysis) to test your figure files. NAAS can convert your figure files to the TIFF file type and meet basic requirements (such as print size, resolution), or provide you with a report on issues that do not meet our requirements and that NAAS cannot fix.

After uploading your figures to PLOS’s NAAS tool - https://ngplosjournals.pagemajik.ai/artanalysis, NAAS will process the files provided and display the results in the "Uploaded Files" section of the page as the processing is complete. If the uploaded figures meet our requirements (or NAAS is able to fix the files to meet our requirements), the figure will be marked as "fixed" above. If NAAS is unable to fix the files, a red "failed" label will appear above. When NAAS has confirmed that the figure files meet our requirements, please download the file via the download option, and include these NAAS processed figure files when submitting your revised manuscript.**Reproducibility:**   To enhance the reproducibility of your results, we recommend that authors of applicable studies deposit laboratory protocols in protocols.io, where a protocol can be assigned its own identifier (DOI) such that it can be cited independently in the future. Additionally, PLOS ONE offers an option to publish peer-reviewed clinical study protocols. Read more information on sharing protocols at https://plos.org/protocols?utm_medium=editorial-email&utm_source=authorletters&utm_campaign=protocols

# Author comment: Author response to Decision Letter 1

**DOI:** 10.1371/journal.pdig.0000737.r002

10 Sep 2025

Attachment Submitted filename: Response to reviewers_.docx

# Peer review: Decision Letter 1

**DOI:** 10.1371/journal.pdig.0000737.r003

**Authors:** Aline Lutz de Araujo, Xiangbin Meng

9 Feb 2026

PDIG-D-24-00593R1Digital citizenship for health: Mapping existing platforms for digital, health and civic literacyPLOS Digital Health Dear Dr. Ogundipe, Thank you for submitting your manuscript to PLOS Digital Health. After careful consideration, we feel that it has merit but does not fully meet PLOS Digital Health's publication criteria as it currently stands. Therefore, we invite you to submit a revised version of the manuscript that addresses the points raised during the review process. Please submit your revised manuscript by Mar 11 2026 11:59PM. If you will need more time than this to complete your revisions, please reply to this message or contact the journal office at digitalhealth@plos.org. When you're ready to submit your revision, log on to https://www.editorialmanager.com/pdig/ and select the 'Submissions Needing Revision' folder to locate your manuscript file. Please include the following items when submitting your revised manuscript:* A letter that responds to each point raised by the editor and reviewer(s). You should upload this letter as a separate file labeled 'Response to Reviewers'. This file does not need to include responses to any formatting updates and technical items listed in the 'Journal Requirements' section below.* A marked-up copy of your manuscript that highlights changes made to the original version. You should upload this as a separate file labeled 'Revised Manuscript with Track Changes'.* An unmarked version of your revised paper without tracked changes. You should upload this as a separate file labeled 'Manuscript'. If you would like to make changes to your financial disclosure, competing interests statement, or data availability statement, please make these updates within the submission form at the time of resubmission. Guidelines for resubmitting your figure files are available below the reviewer comments at the end of this letter. We look forward to receiving your revised manuscript. Kind regards, Aline Lutz de AraujoSection EditorPLOS Digital Health Aline Lutz de AraujoSection EditorPLOS Digital Health Leo Anthony CeliEditor-in-ChiefPLOS Digital Healthorcid.org/0000-0001-6712-6626  **Journal Requirements:**   If the reviewer comments include a recommendation to cite specific previously published works, please review and evaluate these publications to determine whether they are relevant and should be cited. There is no requirement to cite these works unless the editor has indicated otherwise.  **Additional Editor Comments (if provided):**   Thank you for submitting the revised version of your manuscript. The topic is timely and relevant, and the study makes a useful contribution by mapping existing platforms related to Digital Citizenship for Health.

Before the manuscript can be considered for acceptance, minor revisions are required, primarily to improve clarity, transparency, and presentation rather than to address fundamental issues with the study design or data.

Please focus your revision on the following points:

1.Methods section:

Clarify the overall study workflow by clearly distinguishing the platform identification (mapping) phase from the evaluation (walkthrough) phase. Please also provide a concise description of who conducted the walkthrough assessments and how the evaluations were performed.

2.Evaluation criteria:

Provide brief operational clarifications for the evaluation criteria (e.g., ethical, trustworthy, humanistic), and clarify that the binary indicators reflect the presence or absence of predefined observable features.

3.Terminology and language:

Ensure consistent use of key terms throughout the manuscript and improve language clarity and academic tone. Professional language polishing is recommended.

4.Discussion and presentation:

Reduce repetition of results in the Discussion and sharpen the interpretation and implications of the findings. Minor formatting improvements to tables and figures may also enhance readability.

These revisions can be addressed without additional data collection. We look forward to receiving a revised manuscript that incorporates these clarifications. [Note: HTML markup is below. Please do not edit.] **Reviewers' Comments:**   Reviewer's Responses to Questions

**Comments to the Author**

1. If the authors have adequately addressed your comments raised in a previous round of review and you feel that this manuscript is now acceptable for publication, you may indicate that here to bypass the “Comments to the Author” section, enter your conflict of interest statement in the “Confidential to Editor” section, and submit your "Accept" recommendation.

Reviewer #3: All comments have been addressed

Reviewer #4: (No Response)

Reviewer #5: (No Response)

**********

2. Does this manuscript meet PLOS Digital Health’s publication criteria? Is the manuscript technically sound, and do the data support the conclusions? The manuscript must describe methodologically and ethically rigorous research with conclusions that are appropriately drawn based on the data presented.

Reviewer #3: Yes

Reviewer #4: (No Response)

Reviewer #5: Partly

**********

3. Has the statistical analysis been performed appropriately and rigorously?

Reviewer #3: Yes

Reviewer #4: (No Response)

Reviewer #5: I don't know

**********

4. Have the authors made all data underlying the findings in their manuscript fully available (please refer to the Data Availability Statement at the start of the manuscript PDF file)?

The PLOS Data policy requires authors to make all data underlying the findings described in their manuscript fully available without restriction, with rare exception. The data should be provided as part of the manuscript or its supporting information, or deposited to a public repository. For example, in addition to summary statistics, the data points behind means, medians and variance measures should be available. If there are restrictions on publicly sharing data—e.g. participant privacy or use of data from a third party—those must be specified.

Reviewer #3: Yes

Reviewer #4: (No Response)

Reviewer #5: Yes

**********

5. Is the manuscript presented in an intelligible fashion and written in standard English?

PLOS Digital Health does not copyedit accepted manuscripts, so the language in submitted articles must be clear, correct, and unambiguous. Any typographical or grammatical errors should be corrected at revision, so please note any specific errors here.

Reviewer #3: Yes

Reviewer #4: (No Response)

Reviewer #5: No

**********

6. Review Comments to the Author

Please use the space provided to explain your answers to the questions above. You may also include additional comments for the author, including concerns about dual publication, research ethics, or publication ethics. (Please upload your review as an attachment if it exceeds 20,000 characters)

Reviewer #3: 1. This assessment relies heavily on the author's interpretation, as noted in the Discussion section. Without a second reviewer or a clear protocol, the data may be influenced by confirmation bias.

2. The paper identifies "Ethical" and "Trustworthy" as important criteria but does not clearly define these terms. The discussion about why some platforms do not meet these criteria lacks detailed information about essential data protection methods, such as encryption and decentralized storage, which are crucial for health-related platforms.

3. While the paper focuses on "young people," it does not discuss what influences this age group to adopt or reject digital tools. Important factors like social influence and digital self-efficacy should be examined.

4. Clearly explain the scoring system for the 13 criteria. Detail what a "pass" or "fail" means for subjective criteria like "Humanistic."

5. If possible, include a second researcher to validate a sample of the platforms to assess agreement. If this is not possible, add a limitations section explaining how bias was reduced.

6. In the Introduction (when discussing young people's digital habits) or in the Discussion (when examining gaps in user engagement. It is suggested to refer from the paper: “Mobile banking adoption: A multi-factorial study on social influence, compatibility, digital self-efficacy, and perceived cost among Generation Z consumers in the United States “. The paper emphasizes "young people" (Generation Z) but lacks research on what drives this group’s behavior. This study examines the adoption of sensitive digital services, such as banking, among Generation Z, which is comparable to the sensitivity of health data. Including this citation will support your claims about why current platforms may not effectively engage youth.

7. The manuscript should enhance its discussion of Generation Z's behavior, security and privacy methods in digital health, and what "trustworthy" systems look like.Expand on what "Trustworthy" and "Ethical" mean for platforms. Currently, the paper states that they lack security but does not explain what adequate security measures are for health platforms. Discuss modern technologies, such as blockchain and advanced encryption, which are setting standards for protecting digital health data.

8. The paper claims that tools for youth should be "user-friendly." Incorporate research that analyzes how Generation Z adopts technology. Address factors such as social influence and perceived costs or ease of use to explain why current platforms may struggle to engage them.

9. Provide a clear, step-by-step explanation of the walkthrough process. For instance, say that "the researcher registered as a user and attempted to access X content."

Reviewer #4: 1、The tables on pages 12-22 are truncated in multiple places, preferably in the same paragraph on the same page, otherwise it will affect the integrity of the information. Suggest ensuring that all columns are fully displayed, or consider uploading the table as supplementary material.

2、Some references lack DOI or access dates. Suggest adopting the citation format required by the journal and supplementing missing DOIs or stable links.

3、The main text does not specify the executor, duration, whether multiple people evaluate or verify the research method, or how to ensure consistency. Suggest providing additional information on the evaluator's background, evaluation process, recording methods, and how to ensure reliability and validity.

4、How many tools were initially found in the search? How to filter step by step according to the ranking to the last 38, it is recommended to briefly explain to enhance credibility. Clarify the screening process and consider using PRISMA flowchart to assist in explanation.

5、Only use in the text“ ✔” Indicating compliance with standards, the number or proportion of tools that meet each standard has not been counted. Suggest adding a concluding paragraph or data to explain the compliance rate of each standard.

6、Suggest explaining in the limitations section the potential biases that AI retrieval may bring and how to reduce them.

7、In the text, "multilingual support" is considered "equitable" and "accessible format" is considered "inclusive", but it is not clearly defined in the methods section.

8、 It is recommended to use "tools/resources", "platforms", and "initiatives" interchangeably in the text.

9、During the discussion, it was mentioned that 'most tools lack multilingual support', but the results section did not explicitly present data support. Suggest citing specific values

10、Suggest adding a 'Digital Health Citizenship framework diagram' to visually display the intersection of digital, health, and civic literacy. If not applicable, please ignore

11、I think most of the tools mentioned in the article are from Europe and America. Should we discuss the problem and countermeasures of the lack of tools in the global South.

12、The abstract states that young people lack the resources to address their unique challenges, but there is no clear definition of these challenges and needs, which makes the direction of the research questions vague.

13、The abstract points out that "most tools lack multilingual support and alternative formats, excluding users with disabilities or limited digital access". This important discovery seems to be expressed as a universal accessibility issue. Please clarify: within your research framework, should this be specifically understood as' failure to meet the specific needs of a diverse subset of young people within the group, such as non-native speakers, disabled youth, and youth from resource poor areas'?

14、In the introduction, you pointed out that the framework aims to empower marginalized groups. However, categorizing 'young people' directly as a 'marginalized group' may be too vague. Do you mean that young people are generally marginalized in the digital health ecosystem, or do you specifically refer to specific subgroups that are disadvantaged due to digital divide, socioeconomic status, disability, or geographical factors?

15、In the introduction, As mentioned earlier, most young people lack the ability to critically evaluate information and protect privacy. The fifth paragraph immediately begins to discuss the necessity of meaningful co creation and participation. There is a logical leap in between. Is "participation in co creation" itself a key capacity building or empowerment process within the DHC framework for young people who currently lack the necessary skills?

16、When discussing digital tools as a solution, the original text uses "anti bullying tools" as an example and refers to it as "enhancing young people's ability to resist harassment". This is different in nature from the "empowering" abilities emphasized earlier, such as critical thinking, active participation, and co creation. So, in the DHC framework, what is the relationship between protective and reactive "response capabilities" and proactive "empowerment capabilities"? Do they belong to different levels or stages of literacy?

17、The discussion section effectively summarizes the "what are the gaps", but there is insufficient exploration of "why do these gaps exist" and "what do these gaps mean". For example, does the widespread lack of multilingual and alternative formats in tools reveal the "competence based assumption" and "language centrism" in current digital health development? Does the current situation of "fragmentation" reflect the failure of interdisciplinary collaboration, or is it the neglect of comprehensive projects by funding mechanisms? Suggest conducting a thorough analysis of these driving factors.

Reviewer #5: This study aims to map and evaluate existing platforms for "Digital Citizenship for Health" (DC4H). The topic is of significant practical importance, particularly in its focus on the needs of young people, aligning well with current trends in the digital health field. The authors have undertaken substantial work in identifying and cataloging resources, collecting data on 38 platforms, and attempting to establish an evaluation framework based on findings from youth consultations.

However, the current version of the manuscript contains serious flaws that prevent it from meeting the standards for publication in terms of scientific rigor and reporting clarity. The most critical issues are concentrated in the Methods section, where the description suffers from structural confusion, omission of key details, and vague definition of criteria, severely undermining the study's credibility and replicability. Furthermore, pervasive inconsistency in terminology and problematic language expression throughout the manuscript further hinder comprehension.

Therefore, I recommend "Major Revision." The authors must undertake a thorough rewrite and clarification of the research methods, coupled with comprehensive language polishing, to enable this potentially valuable study to be fairly assessed and effectively communicated.

**********

7. PLOS authors have the option to publish the peer review history of their article (what does this mean?). If published, this will include your full peer review and any attached files.

**Do you want your identity to be public for this peer review?**   If you choose “no”, your identity will remain anonymous but your review may still be made public.

For information about this choice, including consent withdrawal, please see our Privacy Policy.

Reviewer #3: No

Reviewer #4: No

Reviewer #5: **Yes:**  Chen Huang

**********

[NOTE: If reviewer comments were submitted as an attachment file, they will be attached to this email and accessible via the submission site. Please log into your account, locate the manuscript record, and check for the action link "View Attachments". If this link does not appear, there are no attachment files.] **Figure resubmission:**   While revising your submission, we strongly recommend that you use PLOS’s NAAS tool (https://ngplosjournals.pagemajik.ai/artanalysis) to test your figure files. NAAS can convert your figure files to the TIFF file type and meet basic requirements (such as print size, resolution), or provide you with a report on issues that do not meet our requirements and that NAAS cannot fix.

After uploading your figures to PLOS’s NAAS tool - https://ngplosjournals.pagemajik.ai/artanalysis, NAAS will process the files provided and display the results in the "Uploaded Files" section of the page as the processing is complete. If the uploaded figures meet our requirements (or NAAS is able to fix the files to meet our requirements), the figure will be marked as "fixed" above. If NAAS is unable to fix the files, a red "failed" label will appear above. When NAAS has confirmed that the figure files meet our requirements, please download the file via the download option, and include these NAAS processed figure files when submitting your revised manuscript. **Reproducibility:**   To enhance the reproducibility of your results, we recommend that authors of applicable studies deposit laboratory protocols in protocols.io, where a protocol can be assigned its own identifier (DOI) such that it can be cited independently in the future. Additionally, PLOS ONE offers an option to publish peer-reviewed clinical study protocols. Read more information on sharing protocols at https://plos.org/protocols?utm_medium=editorial-email&utm_source=authorletters&utm_campaign=protocols

Attachment Submitted filename: Manuscript Review Report.doc

# Author comment: Author response to Decision Letter 2

**DOI:** 10.1371/journal.pdig.0000737.r004

7 Mar 2026

Attachment Submitted filename: Response_to_reviewers__auresp_2.docx

# Peer review: Decision Letter 2

**DOI:** 10.1371/journal.pdig.0000737.r005

**Authors:** Aline Lutz de Araujo, Xiangbin Meng, Lawrence D Hayes

8 Jun 2026

Digital citizenship for health: Mapping and evaluation of existing platforms supporting digital, health, and civic literacy among young people

PDIG-D-24-00593R2

Dear Ogundipe

We are pleased to inform you that your manuscript 'Digital citizenship for health: Mapping and evaluation of existing platforms supporting digital, health, and civic literacy among young people' has been provisionally accepted for publication in PLOS Digital Health.

Before your manuscript can be formally accepted you will need to complete some formatting changes, which you will receive in a follow-up email from a member of our team.

Please note that your manuscript will not be scheduled for publication until you have made the required changes, so a swift response is appreciated.

IMPORTANT: The editorial review process is now complete. PLOS will only permit corrections to spelling, formatting or significant scientific errors from this point onwards. Requests for major changes, or any which affect the scientific understanding of your work, will cause delays to the publication date of your manuscript.

If your institution or institutions have a press office, please notify them about your upcoming paper to help maximize its impact. If they'll be preparing press materials, please inform our press team as soon as possible -- no later than 48 hours after receiving the formal acceptance. Your manuscript will remain under strict press embargo until 2 pm Eastern Time on the date of publication. For more information, please contact digitalhealth@plos.org.

Thank you again for supporting Open Access publishing; we are looking forward to publishing your work in PLOS Digital Health.

Best regards,

Xiangbin Meng, M.D., Ph.D.

Academic Editor

PLOS Digital Health

***********************************************************

**Additional Editor Comments (if provided):**

Thank you for your careful revision of the manuscript. The reviewers’ comments from the previous round have been adequately addressed, and the manuscript has been substantially improved in clarity, structure, and overall presentation. The study is timely, methodologically sound, and makes a valuable contribution to the field. I am pleased to support acceptance of the manuscript for publication.

**Reviewer Comments (if any, and for reference):**

Reviewer's Responses to Questions

**Comments to the Author**

1. If the authors have adequately addressed your comments raised in a previous round of review and you feel that this manuscript is now acceptable for publication, you may indicate that here to bypass the “Comments to the Author” section, enter your conflict of interest statement in the “Confidential to Editor” section, and submit your "Accept" recommendation.

Reviewer #3: All comments have been addressed

Reviewer #4: All comments have been addressed

Reviewer #5: All comments have been addressed

**********

2. Does this manuscript meet PLOS Digital Health’s publication criteria? Is the manuscript technically sound, and do the data support the conclusions? The manuscript must describe methodologically and ethically rigorous research with conclusions that are appropriately drawn based on the data presented.

Reviewer #3: Yes

Reviewer #4: Yes

Reviewer #5: Yes

**********

3. Has the statistical analysis been performed appropriately and rigorously?

Reviewer #3: Yes

Reviewer #4: Yes

Reviewer #5: I don't know

**********

4. Have the authors made all data underlying the findings in their manuscript fully available (please refer to the Data Availability Statement at the start of the manuscript PDF file)?

The PLOS Data policy requires authors to make all data underlying the findings described in their manuscript fully available without restriction, with rare exception. The data should be provided as part of the manuscript or its supporting information, or deposited to a public repository. For example, in addition to summary statistics, the data points behind means, medians and variance measures should be available. If there are restrictions on publicly sharing data—e.g. participant privacy or use of data from a third party—those must be specified.

Reviewer #3: Yes

Reviewer #4: Yes

Reviewer #5: Yes

**********

5. Is the manuscript presented in an intelligible fashion and written in standard English?

PLOS Digital Health does not copyedit accepted manuscripts, so the language in submitted articles must be clear, correct, and unambiguous. Any typographical or grammatical errors should be corrected at revision, so please note any specific errors here.

Reviewer #3: Yes

Reviewer #4: Yes

Reviewer #5: Yes

**********

6. Review Comments to the Author

Please use the space provided to explain your answers to the questions above. You may also include additional comments for the author, including concerns about dual publication, research ethics, or publication ethics. (Please upload your review as an attachment if it exceeds 20,000 characters)

Reviewer #3: No recommendations further

Reviewer #4: For the first time, this study systematically introduces "Civil Literacy" into the digital health assessment framework, breaking through the limitations of traditional focus on functional operations. After modification, the method section has been divided into two stages: platform mapping and platform evaluation, and the structure has been improved.

1、The use of AI assisted recognition platforms is an innovative move, but it also introduces the risk of bias. Suggest providing a more detailed explanation on how to reduce selection bias caused by AI through manual review and triangulation. For example, was a specific prompt word used or was AI recommended based on a specific literature list?

2、Although using a binary rating system of "yes/no" simplifies the evaluation, it also loses a lot of information. For example, a platform with only an English interface and a platform that supports 10 languages were both rated as' fairness=no ', which does not reflect the difference between them. It is suggested to consider introducing a more detailed scoring scale (such as 0-2 or 0-3) and specifying the specific criteria for different scores in the definition (e.g. 0=no multilingual support; 1=support for 2-5 languages; 2=support for more than 5 languages) to enhance the discrimination and sensitivity of the evaluation.

3、When it comes to the issue of fragmentation, we can explore deeper reasons: are there disciplinary barriers (public health, computer science, and education are independent of each other)? Is the sponsor inclined to support projects in a single field? Or is it due to insufficient understanding of the DC4H integration concept among platform developers?

Reviewer #5: (No Response)

**********

7. PLOS authors have the option to publish the peer review history of their article (what does this mean?). If published, this will include your full peer review and any attached files.

**Do you want your identity to be public for this peer review?**   If you choose “no”, your identity will remain anonymous but your review may still be made public.

For information about this choice, including consent withdrawal, please see our Privacy Policy.

Reviewer #3: No

Reviewer #4: No

Reviewer #5: **Yes:**  Chen Huang

**********
